# Strong optical absorption of a metallic film to induce a lensing effect in the visible region

**DOI:** 10.1038/s41598-019-48938-z

**Published:** 2019-08-27

**Authors:** An-Qing Jiang, Kai-Yan Zang, Er-Tao Hu, Hua-Tian Tu, Lei Xu, Wen-Shuai Ren, Osamu Yoshie, Young-Pak Lee, Yu-Xiang Zheng, Song-You Wang, Hai-Bin Zhao, Jun-Peng Guo, C. Z. Wang, K. M. Ho, David. W. Lynch, Liang-Yao Chen

**Affiliations:** 1 Department of Optical Science and Engineering, Fudan University, Shanghai, China; 2 Graduate School of IPS, Waseda University, Fukuoka, Japan; 3 Department of Physics, Hanyang University, Seoul, Korea; 4 Department of Electrical & Computer Engineering, University of Alabama in Huntsville, Alabama, USA; 5 Department of Physics, Iowa State University, Ames, Iowa USA

**Keywords:** Surfaces, interfaces and thin films, Nanophotonics and plasmonics

## Abstract

In this work, the two-dimensional profile of the light transmission through a prism-like metallic film sample of Au was measured at a wavelength of 632.8 nm in the visible intraband transition region to verify that, beyond the possible mechanisms of overcoming the diffraction limit, a strongly nonuniform optical absorption path length of the light traveling in the metal could induce a lensing effect, thereby narrowing the image of an object. A set of prism-like Au samples with different angles was prepared and experimentally investigated. Due to the nonuniform paths of the light traveling in the Au samples, lens-effect-like phenomena were clearly observed that reduced the imaged size of the beam spot with decreasing light intensity. The experimental measurements presented in the work may provide new insight to better understand the light propagation behavior at a metal/dielectric interface.

## Introduction

According to the conventional Snell’s law established by Snell and Descartes in the first half of the 17th century^1^, light that is incident at an angle θ_1_ will be refracted in the positive direction at an angle θ_2_ at an interface consisting of materials 1 and 2 with refractive indices *n*_1_ and *n*_2_, respectively, such that *n*_1_sinθ_1_ = *n*_2_sinθ_2_. This law is valid for optically transparent dielectric materials in a broad photon energy range and under almost all conditions. However, in recent decades, unusual phenomena have been observed with regard to the light path through an interface consisting of a dielectric material and a metallic structure that has a complex refraction index $\tilde{n}$ ($\tilde{n}$ = *n* + i*k*), where *n* and *k* are the refractive index and extinction coefficient, respectively, resulting in great attention paid to exploring the intrinsic mechanism driving these phenomena, which have potential future applications in many fields^2–6^. One interesting phenomenon studied in the previous literature is called the planar metallic lens or “plasmonic lens” effect, which can be applied to overcome the conventional diffraction limit and bend a light path at an interface in a manner that is not necessarily in accordance with Snell’s law^7–21^.

For example, it has been shown that for a parallel light beam with a typical mercury (Hg) wavelength λ of 365 nm in the near-ultraviolet intraband transition region of Ag incident on a sample containing a nanostructure with dimensions much smaller than the wavelength, an image of the nanostructure can be focused by a planar Ag lens attached to the sample, resulting in a reduced light intensity and a considerably narrowed image of the nanostructure compared with that obtained without using a planar Ag lens^9,10^. Due to possible side effects, such as beam scattering and diffraction induced by the nanostructure, the light beams transmitted through the planar Ag lens (or film) will not be parallel, implying that the path z of a light beam traveling through the Ag lens will depend on the individual light paths, which are nonuniformly distributed in the lens. According to the optical principle^22^, the intensity I of light traveling in a strongly absorbing material such as Ag metal will be reduced by a factor of exp(−αz), where α (α = 4π*k*/λ) is the optical absorption coefficient, i.e., I = I_o_exp(−αz), which will cause the intensity of the light emerging from the planar metal lens to decrease with a nonuniform profile depending on the path z traveled by the light beam in the metal. The light transmitted in the center region of the beam will have the shortest path and, consequently, an intensity higher than that of the light transmitted at side positions, which will have a longer path, possibly resulting in an anomalous planar metal lensing effect, which may narrow the image of the nanostructure.

In this work, therefore, we made an effort to experimentally study these phenomena to verify that, beyond the mechanisms mentioned above, a strongly nonuniform absorption path length of the light traveling in the metal could induce the lensing effect. In this study, we prepared a set of prism-like Au samples with different angles. We measured the transmission profile of the light intensity for a laser beam with a wavelength of 632.8 nm in the visible intraband transition region being transmit through the Au sample. Due to the nonuniform paths of the light travelling in the Au, lens-effect-like phenomena with a reduction in image size reduction and a decrease in the intensity of the beam spot were clearly observed. Thus, the experimental measurements reported in this work may provide new insight to better understand the light propagation behavior occurring at a metal/dielectric interface.

## Results

**Figure 1:**
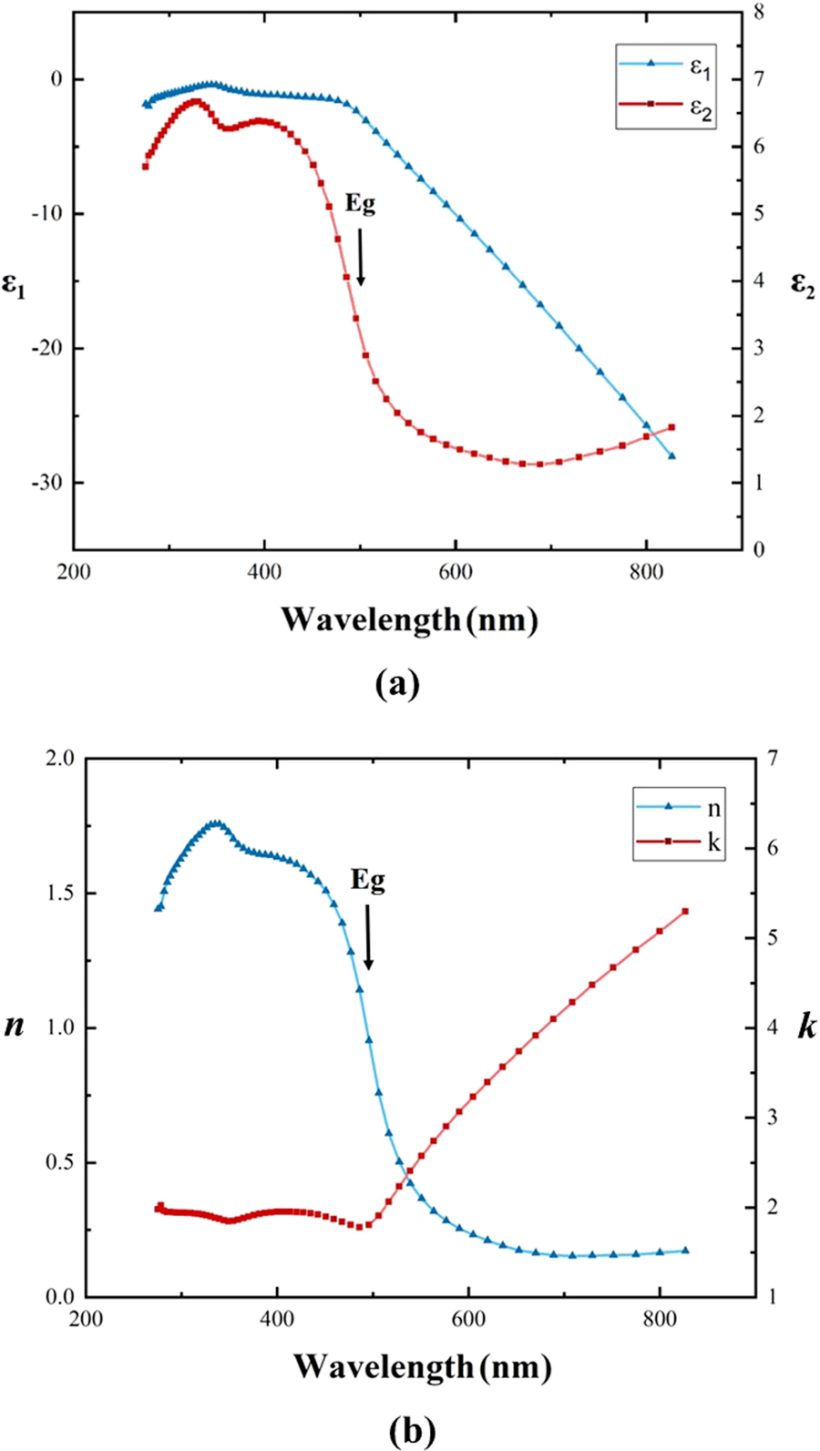
(**a**) Ellipsometrically measured spectra of the real and imaginary parts of the complex dielectric function ε (ε = ε_1_ + iε_2_) of a thick planar Au film sample in the 276–827 nm wavelength range. (**b**) The spectra of the real and imaginary parts, *n* and *k*, of the complex refractive index reduced from the intrinsic relationship to the dielectric function ε. The onset (Eg) of the interband transitions occurs at a photon energy of approximately 2.5 eV, corresponding to a wavelength of approximately 497 nm.

The characteristics of the optical path of light traveling in metal will be affected by many factors, especially the optical constants of the metal, which depend on the sample preparation procedure and conditions^23^. The ellipsometrically measured^24^ spectra of the real and imaginary parts of the complex dielectric function ε (ε = ε_1_ + iε_2_) of a thick planar Au film sample and the spectra of the complex refractive index $\tilde{n}$ reduced from the intrinsic relationship to the dielectric function ε based on $\tilde{n}$ = (ε)^1/2^ are shown in Fig. 1(a,b), respectively, in the wavelength region of 276–827 nm. The real and imaginary parts of the refractive index, *n* and *k*, at a wavelength of 632.8 nm for the Au sample are *n* = 0.193 and *k* = 3.524, respectively. Two types of optical transitions are considered characteristic of the optical properties of Au in the 276–827 nm wavelength region, i.e., the intraband and interband transitions in the 497–827 nm and 276–497 nm wavelength regions, respectively, where the onset (Eg) of the interband transitions occurs at a photon energy of approximately 2.5 eV, corresponding to a wavelength of approximately 497 nm^23–26^.

**Figure 2:**
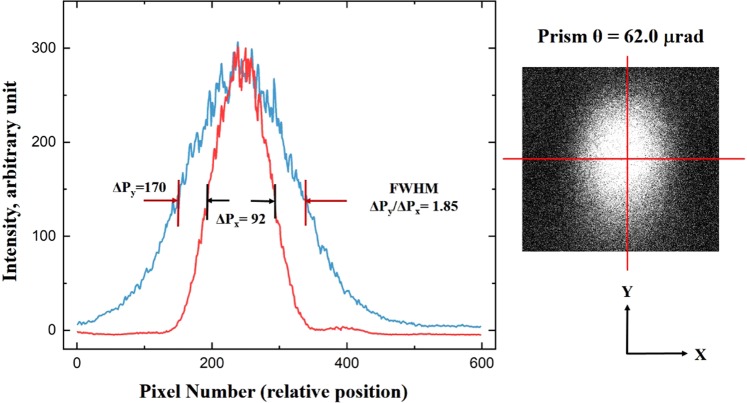
Due to the nonuniform optical path distribution of the light traveling through a Au film with a prism angle of θ = 62.0 μrad along the x direction, the intensity distribution of the full width at half maximum (FWHM) of the light emerging from the sample is narrowed by a factor of approximately 1.85 in the x direction with respect to that in the y direction.

Due to the strong optical absorption of the metal, the intensity distribution will depend on the nonuniform path traveled by the light through the prism-like Au film. As shown in Fig. 2, along the y direction of the sample, the Au film has a uniform thickness. However, the thickness of the film increases linearly with x along the x direction from the thin edge to the thicker side of the prism-like film, causing the path z of the light through the film along the x direction at the edge to be shorter than that on the thicker side of the film, i.e., z ≈ θx under the condition of a small prism angle θ. At a fixed x_i_ position, the light intensity I will have a uniform decay factor because the same amount of photon energy will be absorbed along the y direction, i.e., I = I_o_exp[−αz_y_(x_i_)], where the path z_y_(x_i_) has a constant value that is independent of y. At a fixed y_i_ position, however, the light intensity I decays exponentially because more photon energy is absorbed with increasing x, i.e., I = I_o_exp[−αz_x_(y_i_)], where the path has the form z_x_(y_i_) ≈ θx. Figure 2 clearly shows that for the typical Au film with a prism angle of θ = 62.0 μrad, the numbers of pixels ΔP_x_ and ΔP_y_ counted along the x and y directions to represent the distribution of the full width at half maximum (FWHM) of the light intensity with respect to the center of the image are approximately 92 and 170, respectively. This may explain the phenomenon seen in Fig. 2 that the FWHM lines of the light beam image emerging from the sample are narrowed by a factor (ΔP_y_/ΔP_x_) of approximately 1.85 in the x direction with respect to that in the y direction without the image-narrowing effect.

**Figure 3:**
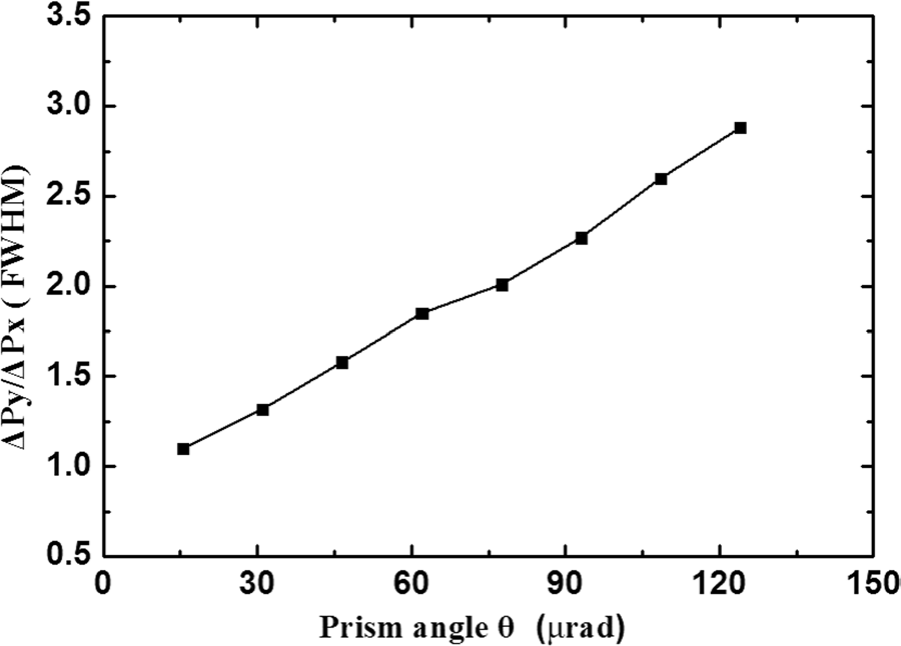
For samples with prism angles ranging from 15.5 μrad to 124.0 μrad, the images (FWHM) of the light beam spots are narrowed by a factor (ΔP_y_/ΔP_x_) that increases from approximately 1.1 to 2.88 in the x direction with respect to the y direction without the metallic lensing effect.

Attributed to the nonuniform optical path distribution of the light transmitted through the prism-like Au film sample along the x direction, the phenomenon of the narrowing of the image size of the light beam spot depending on the prism angle θ was systematically analyzed for all samples, and the results for the narrowing factor are shown in Fig. 3. For the samples with prism angles ranging from 15.5 μrad to 124.0 μrad as mentioned above, the images (FWHM) of the light beam spots were narrowed by a factor (ΔP_y_/ΔP_x_) that increases from approximately 1.1 to 2.88 in the x direction with respect to the y direction without the metallic lensing effect. At the same time, the light intensity was reduced more significantly for a sample with a higher image-narrowing factor, attributed to the photon energy loss along the longer light path traveled in the Au film, which has high optical absorption properties.

## Discussion

The phenomena observed in this work might be used to explain similar metallic “lensing” effects reported in the previous literature in which a planar metallic film has been used to narrow the image of an object^7–21^. A light beam may encounter side effects of microscale light scattering and diffraction by the nanostructure, thus causing the paths traveled by the light through a planar metal film to not be uniformly distributed. In Fang’s experiment^10^, an object with a width of 40 nm was imaged by light with a wavelength of 365 nm onto photoresist through a 75-nm-thick spacer consisting of either a single polymethyl methacrylate (PMMA) layer or a combination of a 40-nm-thick PMMA layer and a 35-nm-thick Ag layer. The measured width of the object image on the photoresist without the Ag layer was approximately 321 nm, meaning that considerable light scattering or a large diffraction angle of approximately 1.085 rad occurred for the light traveling along the side path in the spacer layer, for which the path length would be approximately 2.1 times longer than the path length for the light traveling along the straightforward central direction. With the hybrid spacer consisting of the 40-nm-thick PMMA layer and the 35-nm-thick Ag layer, the width of the object image on the photoresist was narrowed by the Ag lensing effect to approximately 89 nm, corresponding to a narrowing factor of 3.6. Therefore, the light closest to the center of the beam will surely have the shortest path, with an intensity higher than that transmitted at a side position with a longer path subject to more photon energy loss, resulting in the anomalous planar metal lensing effect that narrows the image of the nanostructure by reducing the light intensity, which is simultaneously significantly decreased by the metallic “lensing” effect. In this work, for example, as the light transmits through the Au film (λ = 632.8 nm, *k* = 3.524) with a prism angle of θ = 62.0 μrad at the center of the aperture located at about 1 mm from the thin film edge, the intensity will be decayed by a factor of about 1.3 × 10^−2^.

In summary, an effort has been made in this work to experimentally study and confirm the phenomenon that a nonuniform absorption path distribution for the light traveling in a metallic film will induce a lensing effect that will narrow the image of a light beam spot. A set of prism-like Au samples with different angles were prepared. The transmission profiles of the light intensity were measured for a laser beam with a wavelength of 632.8 nm being transmitted through the Au samples. Due to the nonuniform optical path distribution of the light traveling along the x direction, the phenomenon of the narrowing of the image of the light beam spot depending on the prism angle θ was observed and was systematically analyzed for all samples. The results show that for samples with prism angles ranging from 15.5 μrad to 124.0 μrad, the images (FWHM) of the light beam spots are narrowed by a factor (ΔP_y_/ΔP_x_) that increases from approximately 1.1 to 2.88 in the x direction with respect to the y direction without the lensing effect. At the same time, the anomalous planar metallic lensing effect that narrows the image of the light beam spot also occurs, with the light intensity being significantly decreased with an increasing path length for the light, which is transmitted nonuniformly through the metal. Therefore, the experimental measurements for prism-like Au metal samples reported in this work will provide new insight to better understand the light propagation behavior that occurs at a metal/dielectric interface.

## Methods

### Sample preparation

A series of 8 prism-like Au film samples with a purity of 99.99% were RF (radio frequency) sputtered onto a double-side-polished planar glass substrate in a Leybold-600SP chamber under room temperature conditions. The base pressure was approximately 7 × 10^−6^ mbar, and the film growth rate was approximately 0.128 nm/s, as cross-checked and calibrated within an error of approximately ±2% by means of a Kosaka Surfcorder ET300 and weight measurements. The prism angle θ, which had values of 15.5, 31.0, 45.5, 62.0, 77.5, 93.0, 108.5 and 124.0 μrad, was controlled by means of a microstepper motor in the vacuum chamber to adjust the linear speed of the movement of the mask over the sample *in situ*.

### Experimental setup

**Figure 4:**
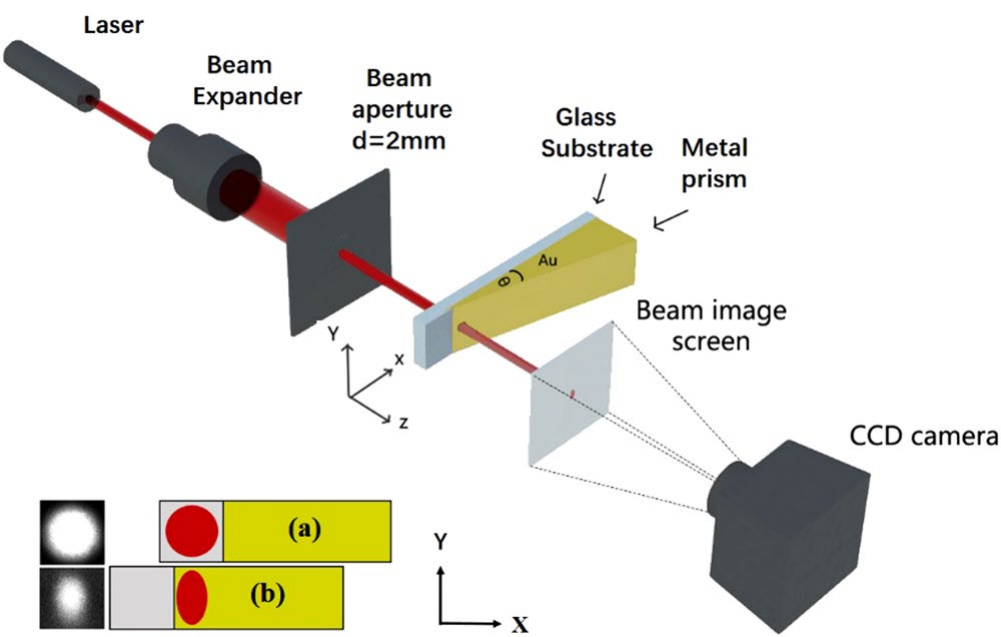
A schematic of the experimental setup used to measure the intensity profiles of a laser beam transmitted through prism-like Au film samples at different x positions. The near-field image of the beam spot (λ = 632.8 nm) that emerged from the sample was displayed on a screen located at a distance of 200 mm from the sample in the z direction. A precision displacement stage was used to move the laser transmission position on the sample (with or without a Au film) in the x direction. The beam spots imaged on the screen were recorded using a CCD camera with 1317 × 1035 pixels, a pixel size of 6.8 × 6.8 μm^2^ and a 12-bit A/D converter resolution. Two typical images are shown, (**a**) for the sample consisting of a glass substrate without a Au film and (**b**) for the Au film sample with a prism angle of 62.0 μrad.

As shown in Fig. 4, a He-Ne laser with a wavelength of 632.8 nm in the visible region was used. The beam deviation was reduced to less than approximately 0.3 mrad by using a 20x beam expander and letting the beam pass through an aperture d of 2 mm in diameter. The laser beam was incident on the glass side of the sample along the z direction, which was normal to the film surface. The size and shape of the beam image were measured using a cooling CCD (charge-coupled device) camera with 1317 × 1035 pixels, a pixel size of 6.8 × 6.8 μm^2^ and a 12-bit A/D converter resolution.

A precision displacement stage was controlled by means of a computer to move the sample along the x direction. The beam image size and shape were quantitatively recorded by the camera as the sample (with or without a Au film) was moved to shift the position through which the laser beam was being transmitted. The near-field intensity profile of the laser beam spot shown on the screen at a z distance of 200 mm from the sample was measured and analyzed to extract the intensity distribution along the x and y directions.
